# College and Hospital Notes

**Published:** 1908-07

**Authors:** 


### COLLEGE AND HOSPITAL NOTES.

---

Columbus Hospital, is the name of a new institution for the care of the sick, built by Dr. Charles R. Borzilleri on Niagara street, Buffalo. The hospital was dedicated June 7, 1908, with appropriate ceremonies. Addresses were made by Mayor Adam, Professor Louis Roversi of New York, Consul Banchetti, the Marquis Alfonso Pappalarde, the Reverend Father Carlo Daglio and Dr. Edward J. Meyer.

The hospital is built and finished after the most approved methods of construction in every detail. Its inside is pure white, its operating room is waterproof, its kitchen, private and semi-private rooms and Dr. Borzilleri's offices are all models of perfect hospital appointments. While it is intended in particular for Italian patients, any sick person of whatsoever nationality will be fortunate to secure a place in this beautifully appointed hospital. Dr. Borzilleri is to be congratulated on conceiving and carrying out to such absolute perfection, an institution that is at once an ornament and a useful addition for the sick and injured of this great city.

---

Dr. Vertner Kenerson, who is going away for a rest, had established, for his own private surgical cases, a small, but very nicely equipped private hospital, at 115 Park street, Buffalo, N. Y. Doctor Kenerson will allow some of his friends to use this very acceptable place for some of their nice cases which they are unable to send to the public hospitals. The nursing is of the best; the furnishings, while convenient for the care of sick people, are comfortable; the prices are $20, $25 and $30 per week.

Special nurse in all severe cases will cost $25 a week in addition to the above rate. The telephone, (which is not listed) is

Frontier 13083, or you can telephone to Dr. Kenerson's office address, which will be found in both telephone books.

---

THE following named members of the graduating class in medicine, University of Buffalo, medical department, 1908, have received hospital appointments as follows:

Douglas P. Arnold, Joseph P. Brennan, Hugh B. Deegan, Karl F. Eschelman, John H. Evans, Joseph A. Gregory, William F. Jacobs, Clayton H. Snover, Fred'k L. Wright, Buffalo General Hospital; Chester C. Cott, Arthur V. Lawler, John F. Ryan, John G. Stowe, Frank A. Valanti, Buffalo Hospital of the Sisters of Charity; Edward Cochrane Gow, Lee Gunn, Buffalo Emergency Hospital; Allen Lee Haenszel, Otto S. McKee, LaVerne Francis Waters, Erie County Hospital; Harry C. Hummell, Robert J. Maichle, St. Mary's Hospital, Rochester, N. Y.; Ray D. Richman, Rochester City Hospital, Rochester, N. Y.; George F. Reusch, German Hospital; Frederick Terrasse, Walter J. M. Wurtz, German Deaconess's Hospital; Benjamin VanCampen, Boston Floating Hospital, Boston, Mass.; Claude C. Williamson, Park Avenue Hospital, Rochester, N. Y.

